# Avoid the Wells

**Published:** 1885-04

**Authors:** 


### AVOID THE WELLS.

The careful examination of water from wells in this city recently made by Dr. Edson and his assistants, revealed facts of great importance which ought to have been discovered and given to the public long ago. It appears that not one of the thousands of wells —driven, artesian, or excavated—that have been made on this island, supplies water that can safely be used in cooking or to quench thirst. Analysis of this water, drawn, it may be, from deposits a thousand feet below the surface, proves that it is heavily charged with organic matter. One can easily believe that water taken by pumps or buckets from ordinary shallow wells from thickly-settled parts of the city is unfit for use, for such a well must have many of the characteristics of a cesspool. Offensive liquids soak through the soil from the surface and enter these receptacles. Sewage inevitably finds its way into them. The water may appear to be clean, and it may be palatable, but it contains the seeds of disease.

Many intelligent persons have supposed, however, that, while shallow open wells might be dangerous, artesian wells and deep driven wells supplied pure water. It is possible to procure pure water in some cities from artesian wells, but, owing to the peculiar geological formation of this island, wells of this kind thousands of feet deep are as bad as those only thirty feet deep. Sewage and other offensive matter constantly accumulating on the surface in a city of 1,400,000 inhabitants works downward, and those who draw water from artesian wells bring it up again. Analysis shows that the deepest wells do not escape contamination.

Many of the most dangerous preventable diseases are caused or communicated by the water that is drunk. Water polluted by matter rejected by the human system should be avoided as one avoids a deadly poison. The water supply of a family or a group of families, if polluted by such matter, may not only cause disease, but also convey it from the sick to those who are in good health. Those who use water taken from such wells as these which Dr. Edson has condemned are always in danger. Asiatic cholera, like some other plagues, is fostered by filth, and it is believed that the disease spreads rapidly in cities where polluted water is used. The Health Department and its agents are engaged in a good work. No one should be allowed to use the water taken from these polluted wells.—*Gailards Medical Journal.*

